# The interplay of autophagy and *β*-Catenin signaling regulates differentiation in acute myeloid leukemia

**DOI:** 10.1038/cddiscovery.2015.31

**Published:** 2015-09-21

**Authors:** K Kühn, C Cott, S Bohler, S Aigal, S Zheng, S Villringer, A Imberty, J Claudinon, W Römer

**Affiliations:** 1 Faculty of Biology, Albert-Ludwigs-University Freiburg, Schänzlestraβe 1, 79104 Freiburg, Germany; 2 BIOSS–Centre for Biological Signalling Studies, Albert-Ludwigs-University Freiburg, Schänzlestraβe 18, 79104 Freiburg, Germany; 3 International Max Planck Research School for Molecular and Cellular Biology (IMPRS-MCB), Max Planck Institute of Immunobiology and Epigenetics, Stübeweg 51, 79108 Freiburg, Germany; 4 Centre de Recherches sur les Macromolécules Végétales (CERMAV), CNRS and Université Grenoble Alpes, 601 rue de la chimie, 38000 Grenoble, France

## Abstract

The major feature of leukemic cells is an arrest of differentiation accompanied by highly active proliferation. In many subtypes of acute myeloid leukemia, these features are mediated by the aberrant Wnt/*β*-Catenin pathway. In our study, we established the lectin LecB as inducer of the differentiation of the acute myeloid leukemia cell line THP-1 and used it for the investigation of the involved processes. During differentiation, functional autophagy and low *β*-Catenin levels were essential. Corresponding to this, a high *β*-Catenin level stabilized proliferation and inhibited autophagy, resulting in low differentiation ability. Initiated by LecB, *β*-Catenin was degraded, autophagy became active and differentiation took place within hours. Remarkably, the reduction of *β*-Catenin sensitized THP-1 cells to the autophagy-stimulating mTOR inhibitors. As downmodulation of E-Cadherin was sufficient to significantly reduce LecB-mediated differentiation, we propose E-Cadherin as a crucial interaction partner in this signaling pathway. Upon LecB treatment, E-Cadherin colocalized with *β*-Catenin and thereby prevented the induction of *β*-Catenin target protein expression and proliferation. That way, our study provides for the first time a link between E-Cadherin, the aberrant Wnt/*β*-Catenin signaling, autophagy and differentiation in acute myeloid leukemia. Importantly, LecB was a valuable tool to elucidate the underlying molecular mechanisms of acute myeloid leukemia pathogenesis and may help to identify novel therapy approaches.

## Introduction

Acute myeloid leukemia (AML) is the most common type of leukemia comprising 80% of the cases in adults and 15–20% in children. It is a disease of the hematopoietic system and is characterized by a massive accumulation of immature and nonfunctional myeloid precursor cells in the blood and the bone marrow.^1^ This arrest of differentiation and infinite proliferation of the myeloid cells are the major problems in the pathophysiology of AML. The initial therapeutic option is still classical chemotherapy with cytarabin and anthracylines that exhibit undesirable side effects especially in older patients. Because of this, the 5-year survival rate is between 30 and 40%, and even more unfavorable in patients >65 years of age.^1,2^ However, AML is quite a heterogeneous disease with variable prognosis, thus classified in subtypes according to morphology and cytogenetics.^3,4^ A high degree of AML cases involve chromosomal translocations that generate chimeric oncoproteins stimulating abnormal proliferation and a block of myeloid differentiation. Common fusion proteins in AML are AML1-ETO, PML-RAR*α* or the MLL translocation.^1,5–7^ Interestingly, all these fusion proteins and the Flt3-internal tandem duplication mutation are associated with aberrant Wnt signaling inducing proliferation and stem cell self-renewal.^8–11^ The induction of Wnt signaling is accompanied by increased *β*-Catenin levels and initiates in turn the expression of tumorigenic Wnt target genes like Cyclin D_1_ or c-myc. Indeed, high *β*-Catenin levels are also found in primary AML samples.^12^ The better understanding of these cytogenetic modifications involves a great potential for the development of personalized and targeted therapy, as the classical chemotherapeutics only tend to highly proliferating cells. The issue of the differentiation arrest is not addressed at all. Recently, impressive successes of a new differentiation therapy concept have been achieved in acute promyelocytic leukemia (APL). In APL, the PML-RAR*α* fusion protein represses – insusceptible to the natural ligand retinoic acid – the transcription of genes important for the myeloid differentiation. Pharmacological doses of all-*trans* retinoic acid (ATRA) or arsen trioxide target specifically the PML-RAR*α* fusion protein and induce its degradation and thereby the transcription and the terminal differentiation of the immature precursor cells. Using this concept, striking remission rates and long-term survival between 80 and 90% could be achieved.^13,14^ In addition, various therapy attempts of ATRA in combination with other chemotherapeutics are in clinical trials to improve the outcome of the classical therapy.^5,15,16^ Unfortunately, ATRA and arsen trioxide are only clinically successful in the rare APL subtype (5–10%) of AML. Thereby, other substances that initiate the differentiation of other AML subtypes are needed and are currently investigated.^17–21^

In our study, we focused on LecB, a fucose-binding lectin of *P. aeruginosa*. Lectins are common carbohydrate-binding proteins produced by animals, plants or microorganisms contributing to cell adhesion and recognition.^22,23^ They are implicated in the innate immune system or act as toxins, for example, Shiga or cholera toxins.^24–26^ The specificity of lectins to a distinct sugar moiety can be used for the recognition of tumor cells as these often exhibit substantial changes in the glycosylation pattern of their surface proteins compared with healthy cells.^27,28^ As fucosylation is often affected in tumors, fucose-specific lectins are of interest as biomarkers and for targeted therapy.^29,30^ Furthermore, it has been shown that LecB is cytotoxic to tumor cells and provokes strong agglutination and an attenuated tumor growth rate.^31,32^

## Results

### LecB induces differentiation and apoptosis of acute monocytic leukemia cells

**Figure 1:**
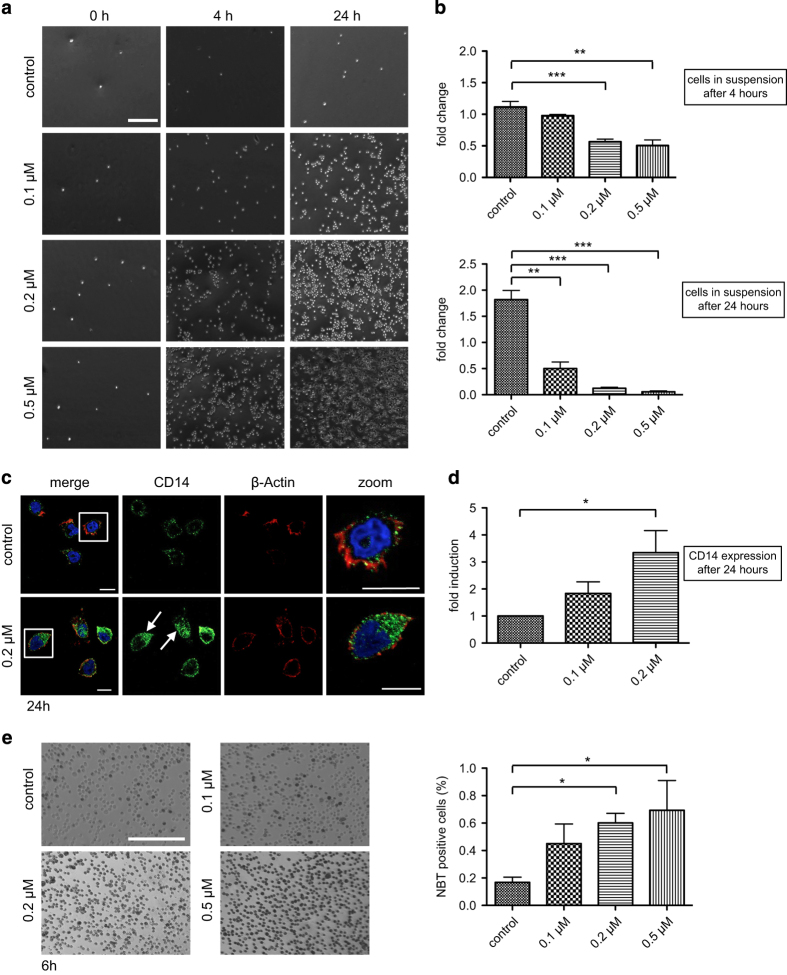
LecB induces differentiation of AML cells. (**a**) Light microscopy images of THP-1 cells treated with the indicated concentrations of LecB. After distinct time points, the cells remaining in suspension were removed and the adhered ones were illustrated. Scale bar, 250 *μ*m. (**b**) The number of cells remaining in suspension upon LecB treatment was determined at indicated time points. Results are expressed as fold change of the cell amount present in the samples relative to the amount at time point 0. (**c**) Immunofluorescence images of THP-1 cells treated with 0.2 *μ*M LecB for 24 h and subsequently stained with a CD14 specific antibody (green, arrows), Phalloidin-Atto-647 (red) and DAPI (blue). Scale bars, 10 *μ*m. (**d**) Flow cytometry analysis of THP-1 cells treated with the indicated LecB concentrations for 24 h revealed a significant induction of CD14 expression. Results are expressed as fold change relative to the nontreated control sample. (**e**) Light microscopy images of THP-1 cells treated with the indicated concentrations of LecB and NBT substrate. After 6 h, the amount of cells bearing the insoluble blue intracellular accumulations were defined as NBT positive and quantified by ImageJ. The values are expressed relative to the total amount of cells. Scale bar, 200 *μ*m. *P<0.05; **P<0.01; ***P<0.001.

**Figure 2:**
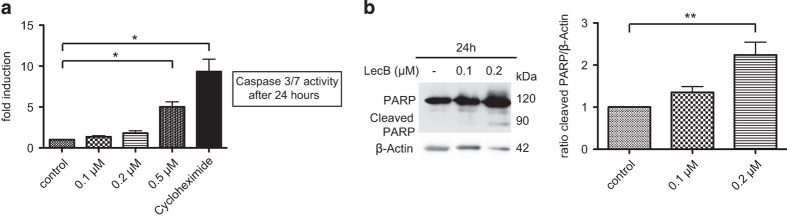
LecB induces apoptosis of AML cells. (**a**) THP-1 cells were treated with indicated concentrations of LecB for 24 h and the induction of Caspase 3/7 activity was determined. Cycloheximide (100 *μ*g/ml) was used as positive control. Results are expressed as fold change relative to the nontreated control sample. (**b**) PARP cleavage was detected by western blot analysis upon LecB treatment for 24 h. The quantification illustrates the fold change of the PARP cleavage normalized to *β*-Actin. Values represent the mean±S.E.M. of at least three independent experiments. *P<0.05; **P<0.01.

First, we examined the role of LecB in acute myeloid leukemia as some lectins have been shown to act as growth inhibiting and cytotoxic on tumor cells.^22^ We administered the lectin at different concentrations (from 0.1 to 0.5 *μ*M) to the monocytic cell line THP-1. Interestingly, LecB was able to induce the differentiation of THP-1 cells as shown by attachment assays, immunophenotyping and the nitrobluetetrazolium (NBT) reduction. When treated with LecB, monocytic THP-1 cells adhered to the cell culture plate in a concentration- and time-dependent manner. After 4 h, cells treated with 0.2 and 0.5 *μ*M LecB attached to a great extent compared with no attachment of nontreated cells (Figure 1a). After 24 h, strong attachment was confirmed for all used concentrations of LecB. In order to quantify the adherence, the amount of cells remaining in suspension upon LecB treatment was determined (Figure 1b). Approximately 56% and 50% of the cells treated with 0.2 and 0.5 *μ*M, respectively, were left in suspension after 4 h. This trend was further validated after 24 h, revealing only 11 and 5% of cells in suspension. Nontreated cells continued to proliferate as usual that was confirmed by an almost doubled cell number after 24 h. Immunophenotyping of CD14 expression, which is upregulated during monocytic differentiation, confirmed the results of the attachment assay. A strong increase in CD14 expression was obvious after 24 h of treatment with 0.2 *μ*M LecB by immunofluorescence (Figure 1c, arrows and zoom). According to this observation, flow cytometry analysis revealed a significant induction (approximately threefold) of CD14 expression (Figure 1d). The differentiation-inducing activity of LecB was also assessed by the NBT reduction assay, an established test that measures the ability of differentiated macrophages to induce a respiratory burst. In the presence of reactive oxygen species, the yellow-colored NBT is converted to a blue insoluble compound inside the cells (Figure 1e). The 6-h incubation with LecB yielded a significant induction of the respiratory burst as 51% (0.2 *μ*M) and 58% (0.5 *μ*M) of the THP-1 cells were NBT positive compared with 15% in the nontreated control (Figure 1e, quantification). When blocking the binding sites of LecB with the soluble ligand L-fucose before the treatment, the cells displayed no differentiation as shown in attachment and NBT reduction assays (Supplementary Figure S1A and B). As it is well established that differentiated cells undergo cell death after a certain lifetime, we assessed the induction of apoptosis. We already observed a hint for apoptosis as the cells treated with 0.5 *μ*M LecB for 24 h seem to change their morphology. The attached cells exhibited membrane blebs and in the following detached again from the cell culture plate after 24 h compared with the 4- h treatment. The cells apparently began to die after they had differentiated. Correspondingly, after 24 h of LecB treatment, the activity of the effector caspases 3 and 7 revealed a significant induction (fivefold) of apoptosis at a concentration of 0.5 *μ*M compared with nontreated cells (Figure 2a). As positive control we used Cycloheximide (100 *μ*g/ml) that is highly toxic for cells because of its inhibitory effect on protein synthesis. Lower LecB concentrations did not induce apoptosis to a high extent. Most probably the apoptosis is delayed corresponding to the slower differentiation. To further elucidate the involvement of apoptosis, we also analyzed poly-(ADP-ribose)-polymerase (PARP) cleavage during LecB treatment. PARP is implicated in the repair of damaged DNA and exhibits a cleavage domain by which caspases can degrade the protein.^33^ In accordance with the caspase results, we observed a twofold increase of the cleaved PARP protein upon stimulation with 0.2 *μ*M LecB for 24 h (Figure 2b). Taken together, LecB induces a very fast differentiation of monocytic THP-1 cells followed by apoptosis of the differentiated cells.

### LecB induces increased autophagy and a reduction of the *β*-Catenin level

**Figure 3:**
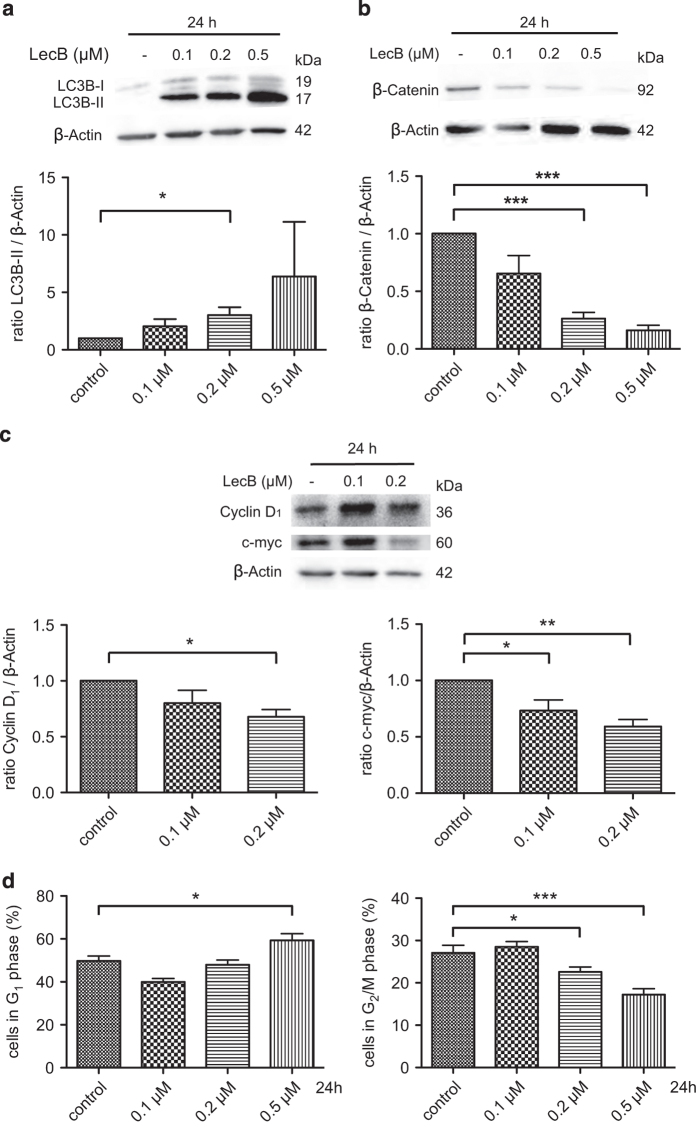
LecB induces autophagy and reduction of *β*-Catenin in AML cells. (**a**) THP-1 cells were stimulated with the indicated concentrations of LecB for 24 h and the conversion of LC3B-I to the autophagosome marker LC3B-II (arrows) was analyzed in western blot experiments. Representative western blot images are illustrated. The quantification shows the fold increase of the LC3B-II normalized to *β*-Actin. (**b**) THP-1 cells were stimulated with LecB for 24 h and the *β*-Catenin protein level was determined in western blot experiments. Representative western blot images and their quantification are illustrated. The results illustrate the fold change of the *β*-Catenin level normalized to *β*-Actin. (**c**) Cells were stimulated with the indicated concentrations of LecB for 24 h and the expression of *β*-Catenin target genes Cyclin D_1_ and c-myc was analyzed in western blot experiments. Representative western blot images are illustrated. The quantification shows the fold change of the protein level normalized to *β*-Actin. (**d**) Treatment with LecB for 24 h affected the proliferation of THP-1 cells shown by cell cycle analysis with propidium iodide and measured by flow cytometry. *P<0.05; **P<0.01; ***P<0.001.

Next, we investigated the mode of action of LecB-mediated differentiation. As the enhanced degradation of redundant proteins is highly important during development, the regular differentiation in the monocyte–macrophage lineage is also accompanied by autophagy.^34^ In addition, the degradation of the oncogenic fusion proteins by autophagy plays a major role in ATRA-induced differentiation^5,13,35^ and in the differentiation process triggered by other substances.^36–39^ During the initiation of autophagy, LC3B gets lipidated and inserts into the autophagosomal membrane (LC3B-II), and for this reason it is considered as a common marker of autophagy. Therefore, we assessed the influence of LecB on autophagy by analyzing the conversion of LC3B-I to LC3B-II in western blot analysis. Indeed, we found a strong increase of LC3B-II in response to rising concentrations of LecB after 24 h (Figure 3a). As a further aspect, we analyzed the role of LecB in *β*-Catenin signaling that is often dysregulated in AML and contributes to stem cell self-renewal, proliferation and also autophagy.^8,12,40–43^ Interestingly, LecB treatment led to a striking concentration-dependent reduction of *β*-Catenin protein levels as depicted in the western blot in Figure 3b. Only 26 and 16% of *β*-Catenin expression was detected after 24 h using 0.2 and 0.5 *μ*M LecB, respectively. Comparable results were observed after 6 h of LecB treatment as the *β*-Catenin level decreased and a conversion of LC3-I to LC3-II was determined (Supplementary Figure S2). In order to check whether *β*-Catenin target genes are affected by LecB treatment, we investigated the expression of Cyclin D_1_ and c-myc. Western blot experiments after 24 h of LecB treatment (0.2 *μ*M) actually confirmed reduced protein levels of Cyclin D_1_ and c-myc by 30 and 40%, respectively (Figure 3c). As differentiated cells lose their capacity to divide and our results point toward a reduction of proliferation-related proteins, we performed cell cycle analysis to gain further insights into the status of proliferation upon LecB treatment. The 24-h LecB treatment revealed G_1_ accumulation of 59% of the cells (0.5 *μ*M) compared with 49% of the cells in the control (Figure 3d, left). In addition, we determined a significant reduction of cells in G_2_/M phase with 22% (0.2 *μ*M) and 17% (0.5 *μ*M) compared with 27% in the nontreated control (Figure 3d, right). The amount of cells in S phase remained stable (data not shown). In summary, LecB treatment causes an induction of autophagy and a decrease in the level of *β*-Catenin and its target proteins accompanied by a clear reduction of proliferative-active THP-1 cells.

### Autophagic activity and the *β*-Catenin level regulate the extent of differentiation

**Figure 4:**
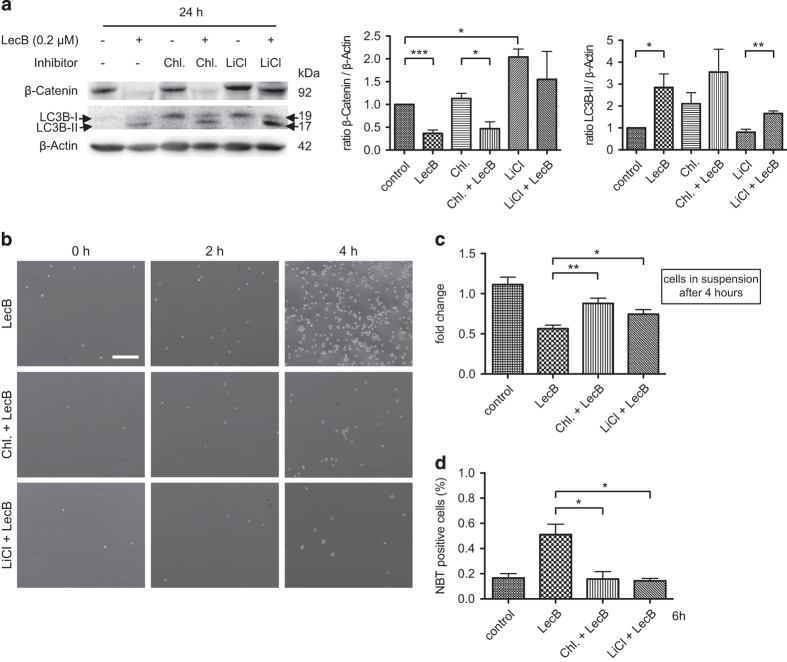
Autophagy and the *β*-Catenin level regulate differentiation. THP-1 cells were treated for 24 h with 0.2 *μ*M LecB±the autophagy inhibitor chloroquine (Chl.: 10 *μ*M) or the GSK-3 inhibitor lithium chloride (LiCl: 20 mM) that stabilized the *β*-Catenin level. (**a**) Western blot analysis and quantification. The shown results illustrate the fold increase of LC3B-II (arrows) and the *β*-Catenin expression normalized to *β*-Actin. (**b**) The graph depicts light microscopy images of THP-1 cells treated with LecB (0.2 *μ*M)±inhibitor for the indicated time points. The inhibitors were capable to counteract LecB-induced attachment to a cell culture plate. Scale bar, 250 *μ*M. (**c**) The number of cells remaining in suspension upon treatment was determined at indicated time points. Results are expressed as fold change of the cell amount present in the samples relative to the amount at time point 0. (**d**) Quantification of an NBT assay of cells treated with the appropriate combinations for 6 h compared with only LecB-treated cells. Values represent the amount of NBT-positive cells relative to the total amount of cells. The results represent the mean±S.E.M. of at least three independent experiments. *P<0.05; **P<0.01; ***P<0.001.

In the following, we aimed at elucidating whether degradation of *β*-Catenin or autophagic activity is the crucial factor for differentiation. Hence, we performed further experiments with chloroquine (10 *μ*M) that inhibits autophagy by preventing the fusion of autophagosomes and lysosomes and the subsequent degradation of autophagic substrates, and lithium chloride (LiCl, 20 mM) that stabilizes the *β*-Catenin protein level by inhibiting the GSK-3*β* kinase activity. We therefore performed western blot analysis after treating the cells with appropriate LecB/inhibitor combinations for 24 h. Using chloroquine alone, the analysis revealed a slight conversion of LC3B-I to LC3B-II and a stable *β*-Catenin level. In combination with LecB (0.2 *μ*M), the *β*-Catenin level significantly decreased to a level comparable to the LecB-treated sample and LC3B-II increased. Even if there was a measurable conversion to LC3B-II in cells treated with LecB/chloroquine, autophagy is supposed to be not functional as only the first steps of autophagy take place whereas the fusion with the lysosome is blocked. In contrast, LiCl provided a stable *β*-Catenin level – even in combination with LecB – and the induction of autophagy was diminished compared with the control samples (Figure 4a). This indicates that a high *β*-Catenin level correlates with low autophagy and vice versa. To verify the functional influence of the inhibitors, we treated cells with LecB in the presence or absence of the inhibitors and performed attachment assays. Cells treated only with chloroquine or LiCl behaved like nontreated cells and proliferated normally (Supplementary Figure S3). Remarkably, LecB-mediated differentiation was attenuated in the presence of chloroquine and LiCl, so that almost no cells attached to the cell culture plate after 4 h of treatment compared with cells treated only with LecB (Figure 4b). The quantification analysis confirmed that inhibition of functional autophagy (chloroquine) and a highly stable *β*-Catenin level (LiCl) significantly diminished the differentiation-inducing effect of LecB as 88% (chloroquine) and 74% (LiCl) of the cells were still in solution compared with 56% in only LecB-treated cells after 4 h (Figure 4c). Furthermore, only 10 and 14% of the cells treated with LecB (0.2 *μ*M) in combination with chloroquine or LiCl were NBT positive compared with 51% of only LecB-treated cells after 6 h (Figure 4d). With regard to proliferation, post 24 h of treatment, there was only mild influence of chloroquine and LiCl on LecB-induced outcomes (data not shown). Summarizing, we showed that both a functional autophagy and a low *β*-Catenin level are crucial for the induction of LecB-mediated differentiation of THP-1 cells.

### The LecB-triggered decrease of *β*-Catenin sensitizes AML cells to mTOR inhibitors

**Figure 5:**
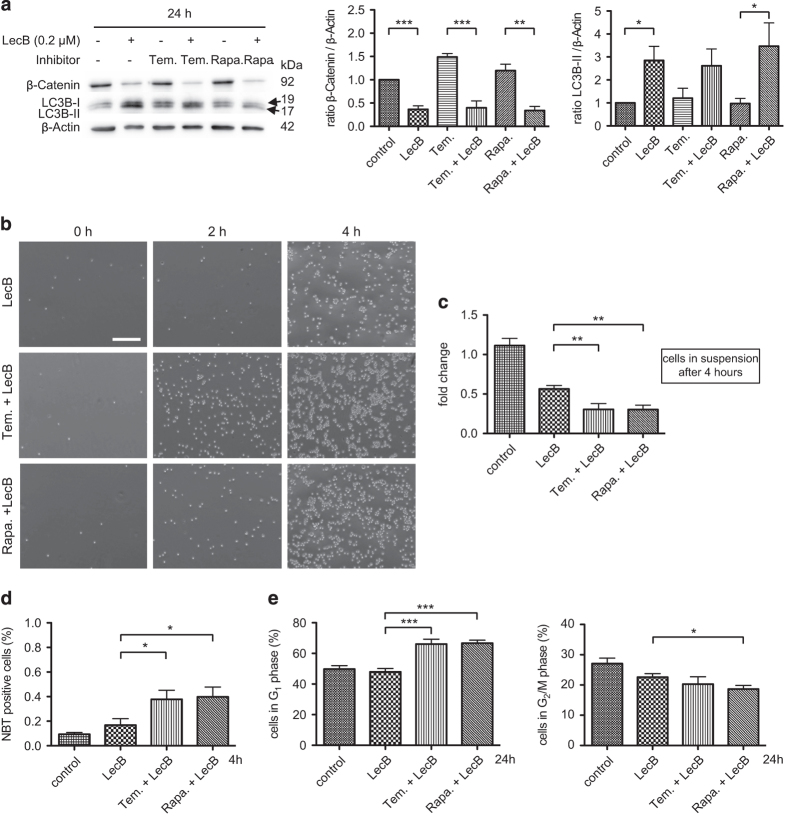
The LecB-triggered decrease of *β*-Catenin sensitizes AML cells to mTOR inhibitors. THP-1 cells were treated with 0.2 *μ*M LecB±mTOR inhibitors Temsirolimus (Tem.: 10 *μ*M) and Rapamycin (Rapa.: 100 nM) for 24 h. (**a**) Western blot analysis and quantification. The shown results illustrate the fold change of the LC3B and the *β*-Catenin expression normalized to *β*-Actin. (**b**) The graph depicts light microscopy images of THP-1 cells treated with LecB±inhibitor. Treatment of THP-1 cells with the mTOR inhibitors and LecB significantly enhanced cell attachment to the culture plate compared with only LecB-treated cells. Scale bar, 250 *μ*m. (**c**) The number of cells remaining in suspension upon treatment was determined at indicated time points. Results are expressed as fold change of the cell amount present in the samples relative to the amount at time point 0. (**d**) Quantification of an NBT assay treated with the appropriate combination for 4 h compared with only LecB-treated cells. Values represent the amount of NBT-positive cells relative to the total amount of cells. (**e**) Cells were treated with LecB in combination with mTOR inhibitors for 24 h and the cell cycle activity was analyzed with propidium iodide and measured by flow cytometry. Values represent the mean±S.E.M. of at least three independent experiments. *P<0.05; **P<0.01; ***P<0.001.

We observed that increased autophagy is essential during differentiation, and thus we investigated the impact of the autophagy-stimulating mTOR inhibitors Temsirolimus (10 *μ*M) and Rapamycin (100 nM). Notably, the treatment with the mTOR inhibitors alone for 24 h did not cause a *β*-Catenin decrease or generation of LC3B-II (Figure 5a). In addition, the mTOR inhibitors did not influence autophagic flux as we observed no larger changes in the level of the autophagic substrate p62 over time (Supplementary Figure S4A). Contrarily, Rapamycin and Temsirolimus were sufficient to largely reduce phosphorylation of the S6-kinase (S6K), another downstream target of the mTOR kinase and implicated in protein synthesis (Supplementary Figure S4B). Thereby, we proved the functionality of the inhibitors at the concentrations used in our study and a specific block of the mTOR-mediated autophagy induction. This finding once more indicates that a stable *β*-Catenin level inhibits autophagy, even if stimulators of autophagy are applied. According to this, mTOR inhibitors were not sufficient to induce any differentiating effect in THP-1 cells (Supplementary Figure S3). Remarkably, by using mTOR inhibitors in combination with LecB (0.2 *μ*M) for 24 h, *β*-Catenin was significantly decreased whereas LC3B-II increased (Figure 5a). Correspondingly, the attachment of cells treated with mTOR inhibitors and LecB was further accelerated compared with only LecB-treated cells (Figure 5b). In particular, only 30% of cells remained in suspension upon treatment with the combination of mTOR inhibitors/LecB for 4 h compared with 56% of the cells treated with LecB only (Figure 5c). The high extent and the speed of differentiation were further approved by NBT reduction as 38% (Temsirolimus/LecB) and 40% (Rapamycin/LecB) of the cells were positive already after 4 h of treatment compared with 17% of cells treated with LecB alone (Figure 5d). Fitting to the remarkable differentiation activity, proliferation was significantly blocked by the mTOR inhibitor/LecB combinations after 24 h. This was shown by a significantly high G_1_ accumulation of ∼67% of the cells compared with 48% of LecB-treated cells and a reduction of cells in the G_2_/M phase with 18% (Rapamycin/LecB) and 20% (Temsirolimus/LecB) compared with 22% in the control (Figure 5e). Notably, the reduction of *β*-Catenin possesses a great potential to sensitize cells to mTOR inhibitors and stimulate autophagy and differentiation.

### LecB strengthens the formation of E-Cadherin/*β*-Catenin complexes

**Figure 6:**
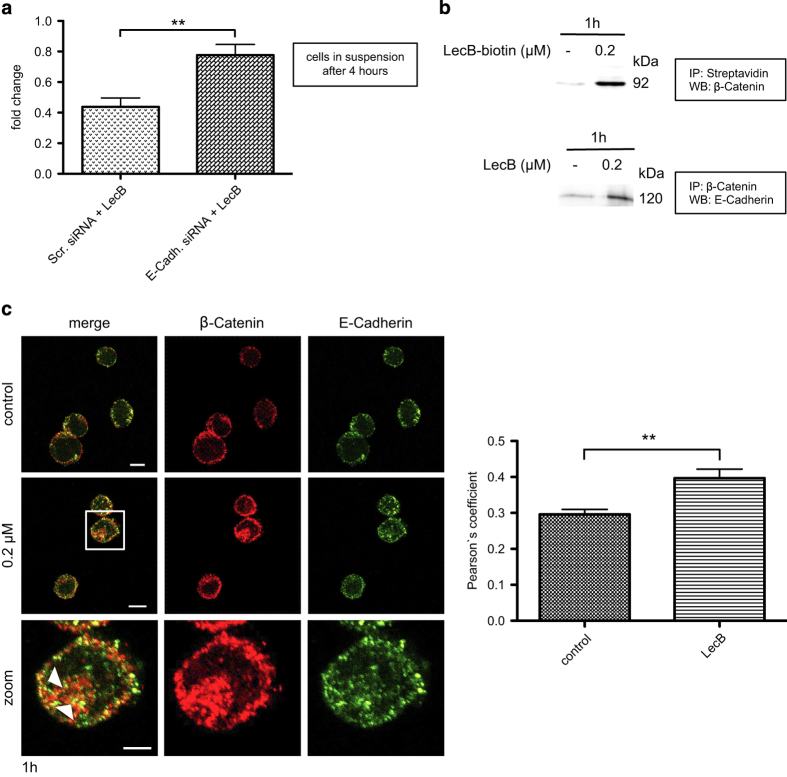
LecB strengthens the formation of E-Cadherin/*β*-Catenin complexes. (**a**) THP-1 cells were first transfected with the indicated siRNAs for 72 h and then treated with 0.2 *μ*M LecB for 4 h. The number of cells remaining in suspension upon the treatment was determined. Results are expressed as fold change of the cell amount present in the samples relative to the amount at time point 0. (**b**) Western blot analysis of THP-1 cells stimulated with 0.2 *μ*M LecB or biotinylated LecB for 1 h. Immunoprecipitations for streptavidin and *β*-Catenin were performed and the precipitated protein complexes were stained for *β*-Catenin and E-Cadherin, respectively. (**c**) Immunofluorescence images of THP-1 cells treated with 0.2 *μ*M LecB for 1 h and subsequently stained with a *β*-Catenin- (green) and an E-Cadherin-specific antibody (red). Colocalizations are indicated with arrowheads. Scale bars, 10 *μ*m (overview) and 5 *μ*m (zoom). Colocalization was determined by Pearson’s coefficient, and the values represent the mean±S.E.M. of 10 analyzed cells per condition. **P<0.01.

In order to determine how the effect of LecB is mediated at the plasma membrane, we analyzed the binding to potential receptor candidates. As E-Cadherin is an important regulator of *β*-Catenin signaling in cancer^44^ and it was reported that it contributes to monocytic differentiation,^45^ we performed immunofluorescence analysis to check for the LecB/E-Cadherin interaction. We observed that LecB binds to the plasma membrane of THP-1 cells and is in close contact with E-Cadherin 30 min after addition of the lectin (Supplementary Figure S5). Next, we investigated whether E-Cadherin has a functional role during LecB-induced differentiation of THP-1 cells. Here, the E-Cadherin protein level was downmodulated to 65% using a specific E-Cadherin siRNA compared with a scrambled control siRNA (Supplementary Figure S6). Interestingly, a reduction of E-Cadherin expression led to significantly diminished LecB-induced differentiation. After 4 h of LecB treatment, 77% of cells transfected with siRNA against E-Cadherin remained in solution compared with only 44% of cells transfected with a scrambled control siRNA (Figure 6a). In addition, we confirmed the interaction between LecB and *β*-Catenin and between *β*-Catenin and E-Cadherin in response to 1 h of LecB treatment by immunoprecipitation (Figure 6b). This was further approved by immunofluorescence images showing pronounced accumulations of E-Cadherin and *β*-Catenin at the plasma membrane and intracellularly (see arrowheads) compared with nontreated cells (Figure 6c). The analysis of these images revealed a significantly increased colocalization of E-Cadherin and *β*-Catenin determined by Pearson’s coefficient. These findings suggest that LecB strengthens the formation of E-Cadherin/*β*-Catenin clusters at the plasma membrane followed by cellular uptake of these complexes, leading to a cell fate different to that of Wnt-induced *β*-Catenin signaling.

## Discussion

The major feature of leukemia cells is a differentiation arrest accompanied by highly active proliferation. A specific and successful differentiation therapy using ATRA and arsen trioxide was established for a small subset of patients carrying the oncogenic PML-RAR*α* fusion protein apparent in APL.^17,46^ As the classical chemotherapeutics exhibit high toxicity and side effects and did not undergo striking changes in the past decades, it is highly important to identify further therapeutics addressing the leukemia cell maturation.

In our study, we identified the fucose-binding lectin LecB as a potent differentiation agent in the AML cell line THP-1. LecB treatment caused a very fast and profound differentiation – even at low concentrations – as shown by adherence assay, immunophenotyping and NBT reduction (Figures 1a–e). These effects were specifically mediated by LecB binding to glycosylated host cell receptors as blocking of the LecB-binding sites with L-fucose prevented the induction of differentiation (Supplementary Figure S1). In addition, the deregulated proliferation was disrupted shown by a remarkable decrease of cells in the G_2_/M phase of the cell cycle and G_1_ accumulation upon lectin treatment (Figure 3d). As differentiated cells usually exhibit a limited lifespan, we confirmed that the cells underwent apoptosis subsequent to their LecB-induced differentiation (Figure 2).

The mechanism of action of LecB involved an induction of autophagy and a decrease of *β*-Catenin (Figures 3 and 4). Actually, an aberrant Wnt/*β*-Catenin signaling, modulated by oncogenic fusion proteins, is frequently implicated in the pathogenesis of leukemia and contributes to stem cell self-renewal, reduced differentiation and apoptosis.^8,47,48^ We found that the *β*-Catenin level decreased whereas the conversion of LC3B-I to the lipidated form LC3B-II increased in response to LecB treatment (Figure 3a). The LecB-induced reduction of *β*-Catenin diminished the expression of proliferation-inducing target genes like Cyclin D_1_ and c-myc and the extent of proliferation (Figures 3b–d). Interestingly, a functional autophagy was essential for the differentiation of AML cells as well, even if the *β*-Catenin level was low, as it was seen for samples treated with chloroquine in the presence and absence of LecB. Hence, the inhibition of autophagy attenuated the LecB-mediated effects on differentiation (Figure 4). Accordingly, mTOR inhibitors, which stimulate autophagy, are already in preclinical and clinical studies. However, the clinical benefit of these dugs either alone or in combination with chemotherapeutics has been relatively low and disappointing.^49–51^ Interestingly, our results indicate that *β*-Catenin stabilization by LiCl attenuates the conversion to LC3B-II regardless of LecB addition (Figure 4). In addition, the application of the autophagy-stimulating mTOR inhibitors Temsirolimus and Rapamycin to THP-1 cells did not induce any differentiation as long as the *β*-Catenin level remained stable (Figure 5). This notably indicates that a high *β*-Catenin level inhibits functional autophagy and compensates the effect of mTOR inhibitors. The inhibitory effect might illustrate an explanation for the resistance of AML patients to mTOR inhibitors.^11,49^

We found two important effects of the high *β*-Catenin expression that might mediate the block of differentiation, namely the induction of target protein expression and the inhibition of autophagy. However, the distinct mechanism by which *β*-Catenin maintains the stem cell-like character of the cancer cells is still controversially discussed and the genetic program is not fully understood. First, it is suggested that *β*-Catenin is able to block differentiation by modulating differentiation- and proliferation-specific genes.^52^ Exemplarily, *β*-Catenin repressed C/EBP*α* expression in adipogenesis.^53^ In addition, the regulators of hematopoietic stem cell self-renewal, Hoxb4 and Bmi1, are target genes of the Wnt/*β*-Catenin signaling.^54^ Second, it was reported in recent literature that *β*-Catenin inhibits autophagy in several cancer cells. Overexpression of *β*-Catenin decreases drug-mediated cytotoxicity and autophagy in breast cancer stem-like cells.^43^ This is fitting to our findings using mTOR inhibitors as the high expression of *β*-Catenin comprised the induction of autophagy as well. It is further proposed that the inhibition is mediated via a negative correlation between *β*-Catenin and Beclin that is part of the autophagy initiation complex.^55,56^

Finally, we observed a remarkable influence of E-Cadherin during the differentiation of THP-1 cells. E-Cadherin is capable to compete with Wnt signaling by sequestering *β*-Catenin at the plasma membrane, mediating cell–cell adhesion and antagonizing the Wnt signaling. Interestingly, E-Cadherin expression is often epigenetically silenced by aberrant hypermethylation, leading to enhanced migration in AML, and is correlated with poor prognosis.^57^ In our study, we confirmed the importance of E-Cadherin for the inhibition of tumor progression as downmodulation of E-Cadherin efficiently attenuated LecB-induced differentiation (Figure 6a). Indeed, we could prove that LecB is in a complex with *β*-Catenin and increased colocalization of E-Cadherin and *β*-Catenin (Figures 6b and c). This argues that LecB interacts with and further promotes the formation of E-Cadherin clusters that stabilize *β*-Catenin firmly in the complex and thereby prevent nuclear translocation and target gene expression as already suggested.^58,59^

Thus, our study provides for the first time a link between E-Cadherin, *β*-Catenin, autophagy and differentiation in AML. Moreover, the lectin LecB was a valuable tool to elucidate the underlying molecular mechanisms of the aberrant Wnt/*β*-Catenin signaling in AML and to gain further insights into possible points of application and new therapeutic approaches.

## Materials and methods

### Cell culture

THP-1 cells were obtained from the BIOSS Toolbox facility at the University of Freiburg (Freiburg, Germany). Cells were maintained in RPMI-1640 supplemented with 10% FBS, 2 mM L-glutamine and 1% Penicillin Streptomycin (Life Technologies, Gibco, Darmstadt, Germany) at 37 °C and 5% CO_2_.

### Pharmacological inhibitors, siRNA and antibodies

Treatment with inhibitors was performed as follows: we used the autophagy inhibitor chloroquine (10 *μ*M), the mTOR inhibitors Temsirolimus (10 *μ*M) and Rapamycin (100 nM) and the GSK-3 inhibitor LiCl (20 mM). Where indicated, cells were preincubated with the inhibitors for 20 min at 37 °C and inhibitors were maintained in the medium during lectin incubation. All inhibitors were purchased from Sigma-Aldrich (Munich, Germany). Preblocking of LecB with 0.3 M L-fucose (Sigma-Aldrich) was performed for 30 min at room temperature, and afterwards the preblocked LecB was added to the cells. E-Cadherin siRNA and scrambled siRNA were purchased from Santa Cruz Biotechnology (Heidelberg, Germany). Antibodies used in this study were purchased from Cell Signaling Technology (Leiden, The Netherlands) and Santa Cruz Biotechnology.

### Differentiation

Cells were treated with the indicated concentrations of LecB and the differentiation was assessed by the attachment to a cell culture plate. Therefore, cells in suspension were removed, attached cells were washed once with PBS and images were acquired using an EVOS microscope (Peqlab Biotechnologie GmbH, Erlangen, Germany). For the quantification, cells remaining in suspension were counted with a hemocytometer and illustrated as fold change compared with the cell amount at the starting time point. The NBT assay is based on the ability of functionally differentiated myelomonocytic cells to induce a respiratory burst. Cells were incubated with indicated concentrations of LecB±inhibitors and NBT for distinct time points. Afterwards, cells containing the blue insoluble dye were counted using ImageJ A 1.45b (NIH, Bethesda, MD, USA). NBT was purchased from Sigma-Aldrich. Differentiation was additionally assessed by immunophenotyping with the monocyte/macrophage marker CD14 in immunofluorescence and FACS experiments. FACS measurements were performed with FACS Gallios (Beckman Coulter, Krefeld, Germany); results were analyzed using FlowJo 8.2 (Treestar Ashland, OR, USA).

### Cell cycle analysis

Cells were treated with indicated concentrations of LecB±inhibitors for 24 h, and then cell nuclei were isolated and stained using the CycleTEST PLUS DNA Reagent Kit (BD Biosciences, Heidelberg, Germany) according to the manufacturer’s protocol and FACS measurements were performed.

### Apoptosis analysis

In order to detect the apoptotic activity of cells upon LecB treatment, we treated the cells with indicated concentrations of LecB for 24 h and assessed the Caspase 3/7 activities with the Caspase-Glo 3/7 assay (Promega, Mannheim, Germany) according to the manufacturer’s instructions. In addition, we analyzed PARP cleavage that is typically induced by caspases during extensive DNA damage. Cycloheximide (100 *μ*g/ml) was used as positive control.

### Transfection

Cells were transfected with specific siRNA using Lipofectamine 2000 (Life Technologies, Invitrogen, Darmstadt, Germany) according to the manufacturer’s protocol prepared in OptiMem medium (Life Technologies, Gibco, Darmstadt, Germany). Cells were incubated at 37 °C, washed after 6 h of transfection and incubated with supplemented medium for another 66 h. Afterwards, lectin treatment was performed.

### Western blot analysis

Cells were treated with LecB for indicated time points, washed with PBS and lysed in RIPA buffer supplemented with protease and phosphatase inhibitors. Protein concentration was analyzed using the Pierce BCA protein assay kit (Thermo Scientific, Schwerte, Germany) and measured with Tecan Safire microplate reader (Tecan, Crailsheim, Germany) using Magellan software. Next, 5–20 *μ*g of the protein lysates were separated by SDS-PAGE and transferred to a nitrocellulose membrane. The membrane was blocked with 3% BSA for 1 h and incubated with primary and HRP-linked secondary antibodies for 1 h each. Detection was performed by a chemiluminescence reaction using the Fusion-FX7 Advance imaging system (Vilber-Lourmat, Eberhardzell, Germany).

### Immunofluorescence and FACS analysis

Cells for immunofluorescence and FACS analysis were incubated±0.2 *μ*M lectin at 37 °C for 24 h and fixed afterwards with 4% PFA for 15 min at room temperature. All following steps were performed at room temperature. For antibody staining, the samples were treated with ammonium chloride solution (50 mM) and saponin/BSA solution for 15 min each. Next, the cells were incubated for 30 min with the primary (1 : 100) and the labeled secondary antibody (1 : 200), respectively, and FACS analysis was performed as described above. Immunofluorescence samples were additionally washed, stained with DAPI (Roth, Karlsruhe, Germany) and Phalloidin-Atto-647 (Sigma-Aldrich) for 10 min and mounted in Mowiol 4-88 (Roth). Fluorescence images were recorded at room temperature using a laser-scanning confocal fluorescence microscope system (Nikon Eclipse Ti-E inverted microscope equipped with a Nikon A1R confocal laser scanning system, 60× oil immersion objective, NA=1,49, laser lines: 405, 488, 561 and 640 nm, Nikon GmbH, Düsseldorf, Germany). Image acquisition and analysis was performed with NIS-Elements (Nikon, version 4.10.04), and for quantification of colocalizations, Pearson’s coefficient was determined.

### Statistical analysis

Data of at least three independent experiments are presented as mean±S.E.M. and were compared by unpaired *t*-test or the one-sample *t*-test using GraphPad Prism 5.0 (La Jolla, CA, USA). A *P-*value of ≤0.05 was considered statistically significant (*P<0.05; **P<0.01; ***P<0.001). Values represent the mean±S.E.M. of at least three independent experiments.

## Abbreviations

AML: acute myeloid leukemia
APL: acute promyelocytic leukemia
ATRA: all-*trans* retinoic acid
LiCl: lithium chloride
NBT: nitrobluetetrazolium
PARP: poly-(ADP-ribose)-polymerase.
