# Supplementary material for: The interplay of autophagy and β-Catenin signaling regulates differentiation in acute myeloid leukemia
Source: Cell Death Discov. 2015 Sep 21;1:15031–. doi: 10.1038/cddiscovery.2015.31 (PMC4979480; doi:10.1038/cddiscovery.2015.31)
Supplement: Supplementary Figures [file cddiscovery201531-s1.pdf]

The interplay of autophagy and β-Catenin signaling

regulates differentiation in acute myeloid leukemia

Katja Kühn[1,2], Catherine Cott[1,2], Sheila Bohler[1,2,#], Sahaja Aigal[1,2,3], Shuangshuang Zheng[1,2], Sarah Villringer[1,2], Anne Imberty[4], Julie Claudinon[1,2] and Winfried Römer[1,2,*]

Supplemental Figures

The additional figures provide further results in order to support the major conclusions shown in the main text.

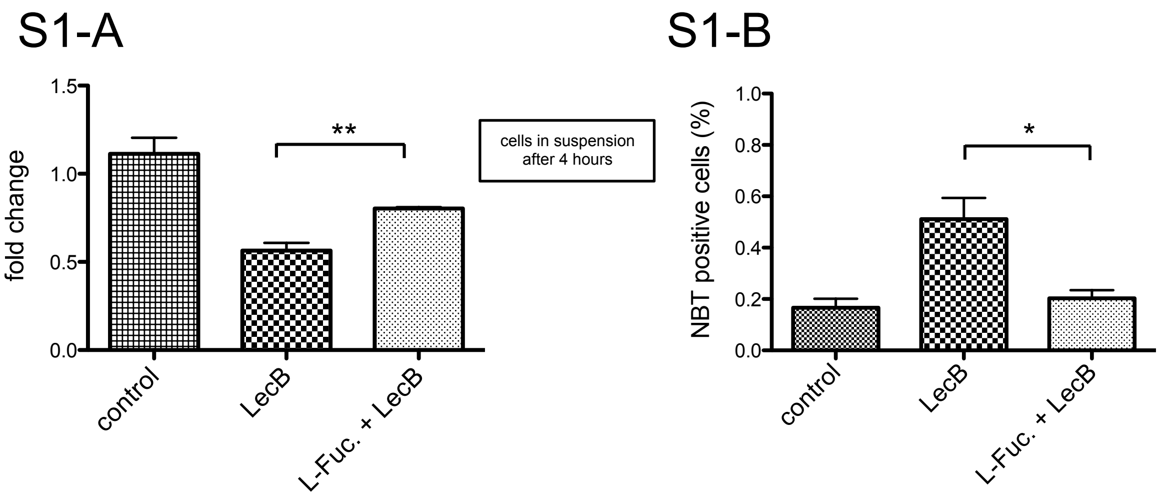

Supplemental Figure 1. Fucose blocked the LecB–mediated differentiation.
(A) The number of cells, remaining in suspension upon 0.2 μM LecB treatment ± pre-incubation with 0.3 M L-fucose was determined after 4 hours. Results are expressed as fold change of the cell amount present in the samples relative to the amount at time point 0. (B) Cells were treated with LecB ± pre-treatment with 0.3 M L-fucose for 6 hours; the blue cells were quantified as NBT-positive. The assay showed significantly lower amount of NBT-positive cells. Values represent the mean ± SEM of at least three independent experiments.

S2

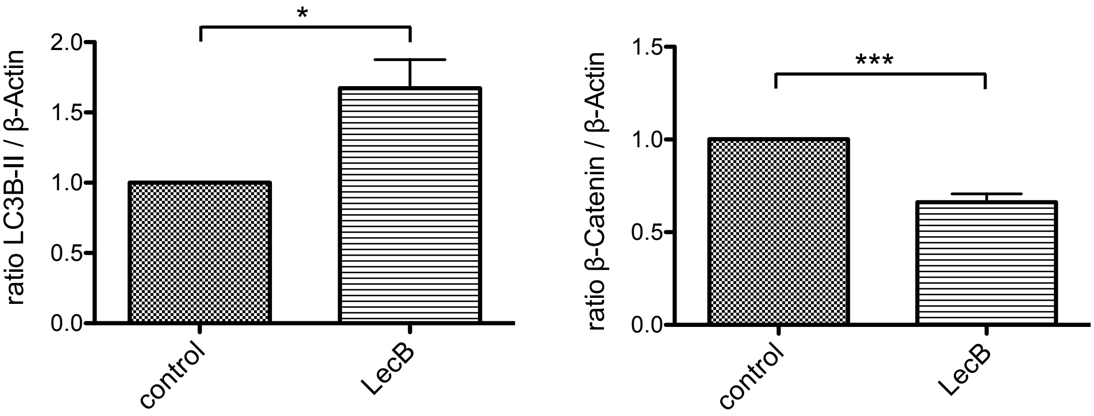

Supplemental Figure 2. LecB induces autophagy and reduction of β-Catenin in AML cells. THP-1 cells were treated with 0.2 μM LecB for 6 hours and Western Blot analysis was performed. The shown results illustrate the fold change of the conversion of LC3B-I to LC3B-II and the β-Catenin expression normalized to β-Actin.

S3

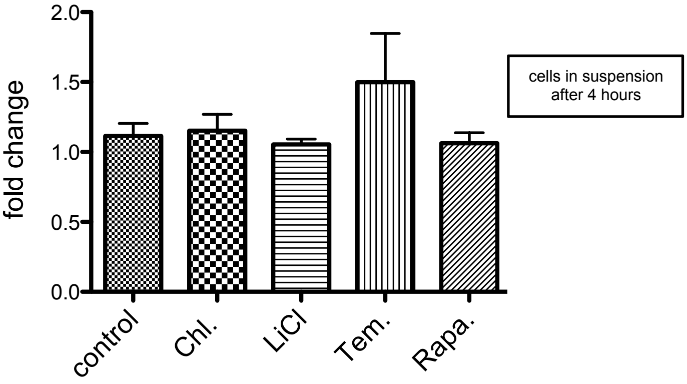

Supplemental Figure 3. Inhibitors alone did not induce any differentiation.

Cells were treated with the indicated inhibitors and the number of cells remaining in suspension was determined at indicated time points. Results are expressed as fold change of the cell amount present in the samples relative to the amount at time point 0. Values represent the mean ± SEM of at least three independent experiments.

S4-A

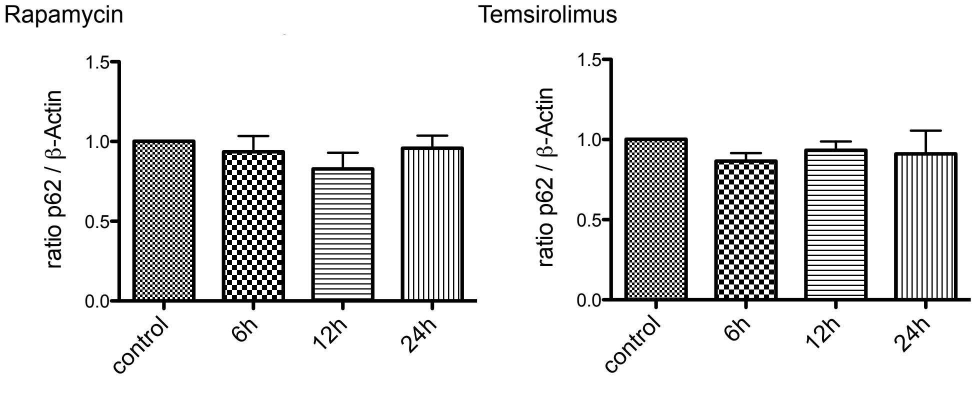

S4-B

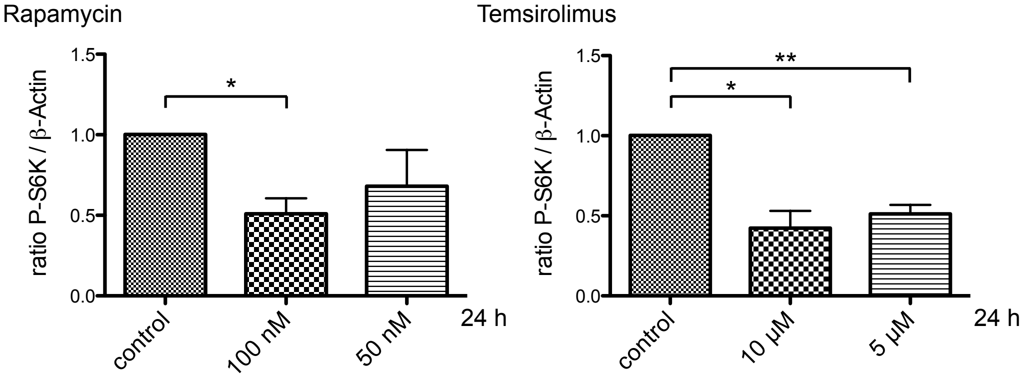

Supplemental Figure 4. The mTOR inhibitors Rapamycin and Temsirolimus reduce phosphorylation of the mTOR target S6-kinase, but have no effect on the autophagic substrate p62.

(A) THP-1 cells were treated with the mTOR inhibitors Rapamycin (100 nM) and Temsirolimus (10 µM) for the indicated time points and the protein level of the autophagic substrate p62 was analyzed. The shown results illustrate the fold change of the p62 level normalized to β-Actin. (B) THP-1 cells were treated with the mTOR inhibitors Rapamycin (100 and 50 nM) and Temsirolimus (10 and 5 µM) for 24 hours and the phosphorylation of the S6K (Thr 389) was analyzed. The shown results illustrate the fold change of the P-S6K level normalized to β-Actin. Values represent the mean ± SEM of three independent experiments.

S5

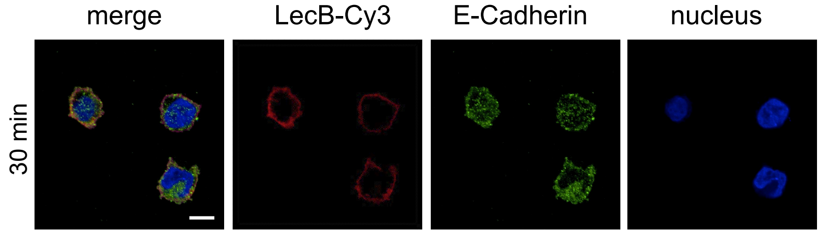

Supplemental Figure 5. E-Cadherin and LecB are in close contact at the cell membrane. Immunofluorescence images of THP-1 cells stimulated with Cy-3-labeled LecB (red) for 30 minutes and stained with an E-Cadherin-specific antibody (green) and DAPI (blue). Scale bar is equal to 10 μm.

S6

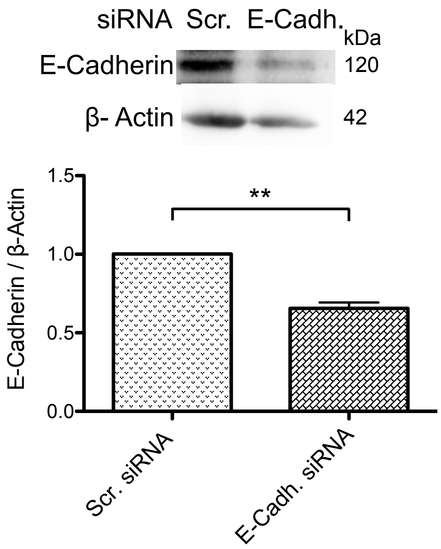

Supplemental Figure 6. E-Cadherin was down-modulated using a specific siRNA. Cells were transfected with a specific E-Cadherin siRNA and a scrambled control siRNA for 72 hours. The down-modulation of E-Cadherin was confirmed by Western Blot analysis. The values shown in the quantification illustrate the fold change of the protein level normalized to β-Actin. Values represent the mean ± SEM of three independent experiments.
